# Effects of Perineal Priming and Perineal Support on Perineal Trauma and the Duration of the Second Stage of Labor Among Primigravidae

**DOI:** 10.7759/cureus.113495

**Published:** 2026-07-28

**Authors:** Farheen Khan, Himanshu Vyas, Suresh K Sharma

**Affiliations:** 1 Radiation Oncology, All India Institute of Medical Sciences, Gorakhpur, Gorakhpur, IND; 2 College of Nursing, All India Institute of Medical Sciences, Jodhpur, Jodhpur, IND

**Keywords:** perineal priming, perineal tear, perineal trauma, primi-gravidae, second stage of labor

## Abstract

Introduction: This study aimed to investigate the effects of perineal priming and perineal support on perineal trauma and the duration of the second stage of labor among primigravidae.

Methods: A quantitative research approach with a posttest-only design was chosen, and 40 primigravidae were assigned to each of the experimental and control groups. Primigravidae in the experimental group received perineal priming and perineal support, whereas those in the control group received routine care during labor. The frequency of perineal trauma and the duration of the second stage of labor were compared.

Results: There were no statistically significant differences between the groups in perineal trauma or the duration of the second stage of labor at the 0.05 level.

Discussion: Perineal priming and perineal support are simple, low-cost interventions that are potentially feasible in routine maternity care. They are also regarded as innovative because they include various interventions that have not previously been combined. Thus, the decision to use these interventions could be based on the midwife’s knowledge and the mother’s willingness.

## Introduction

Perineal trauma, which includes spontaneous perineal tears and episiotomy, is one of the most common maternal complications of vaginal birth. It is associated with postpartum pain, hemorrhage, infection, delayed wound healing, dyspareunia, fecal incontinence, and reduced quality of life [1-6]. Worldwide, the incidence of episiotomy ranges from less than 10% in several high-income countries to more than 70% in some low- and middle-income countries [7,8]. In India, episiotomy rates range from 40% to 90%, particularly among primigravidae, reflecting its continued routine practice in healthcare settings [9,10]. Perineal priming, however, is a simple, non-invasive intervention that aims to improve perineal elasticity. It also relaxes the muscles, which helps in stretching the vaginal outlet during delivery. Perineal priming consists of several distinct components, including perineal massage and warm compresses, performed in a systematic manner. In addition, perineal support during crowning helps control fetal head expulsion, minimizes excessive perineal stretching, and further reduces the risk of anal sphincter injuries [11-17]. Therefore, the present study was undertaken to assess the effectiveness of a combined approach of perineal priming and perineal support in reducing perineal trauma and the duration of the second stage of labor among primigravidae.

## Materials and methods

A quantitative research study was conducted using a quasi-experimental posttest-only control group design among primigravidae delivering at All India Institute of Medical Sciences, Jodhpur, who consented to participate during the data collection period. The sample size was calculated using the prevalence rate of perineal trauma, which was 35 in each of the experimental and control groups, respectively, and increased to 40 in each group after accounting for standard error and a 10% dropout rate. A non-probability total enumerative sampling technique was used, and all primigravidae meeting the inclusion criteria (≥37 weeks of gestation) were selected, while those with high-risk conditions such as diabetes, hypertension, heart disease, kidney disease, and autoimmune disorders were excluded. Participants were sequentially allocated to the control group first and then to the experimental group. The study included seven sociodemographic variables (age, educational status, religion, induction of labor, BMI, fetal weight, and type of family), with perineal priming and support as the independent variable, and perineal trauma and duration of the second stage of labor as the outcome variables.

**Figure 1:**
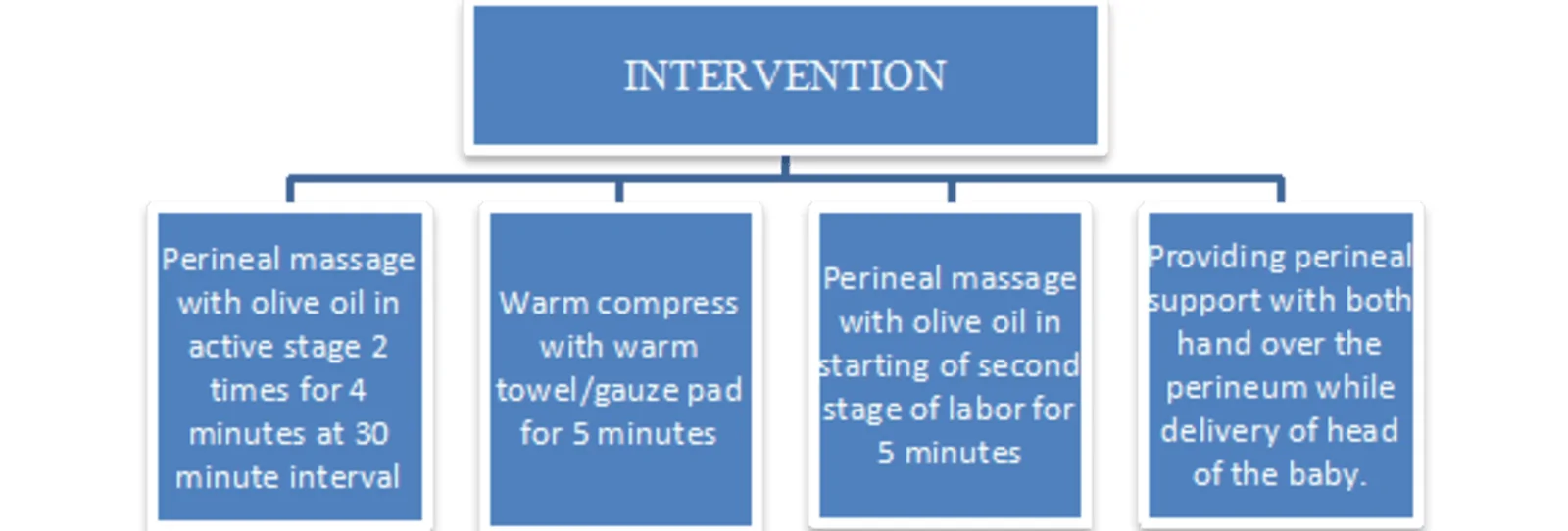
Perineal priming. Components of the intervention implemented during labor.

The experimental group received perineal priming and support during the first stage of labor and the early second stage of labor, while the control group received standard care, which included perineal support at the time of fetal head expulsion. Data were collected using a sociodemographic proforma and a perineal trauma assessment tool covering episiotomy and various degrees of perineal tears. Tool reliability was established through inter-rater reliability (0.90), and validity was confirmed by expert opinion. The process of perineal priming is shown in Figure 1. 

**Figure 2:**
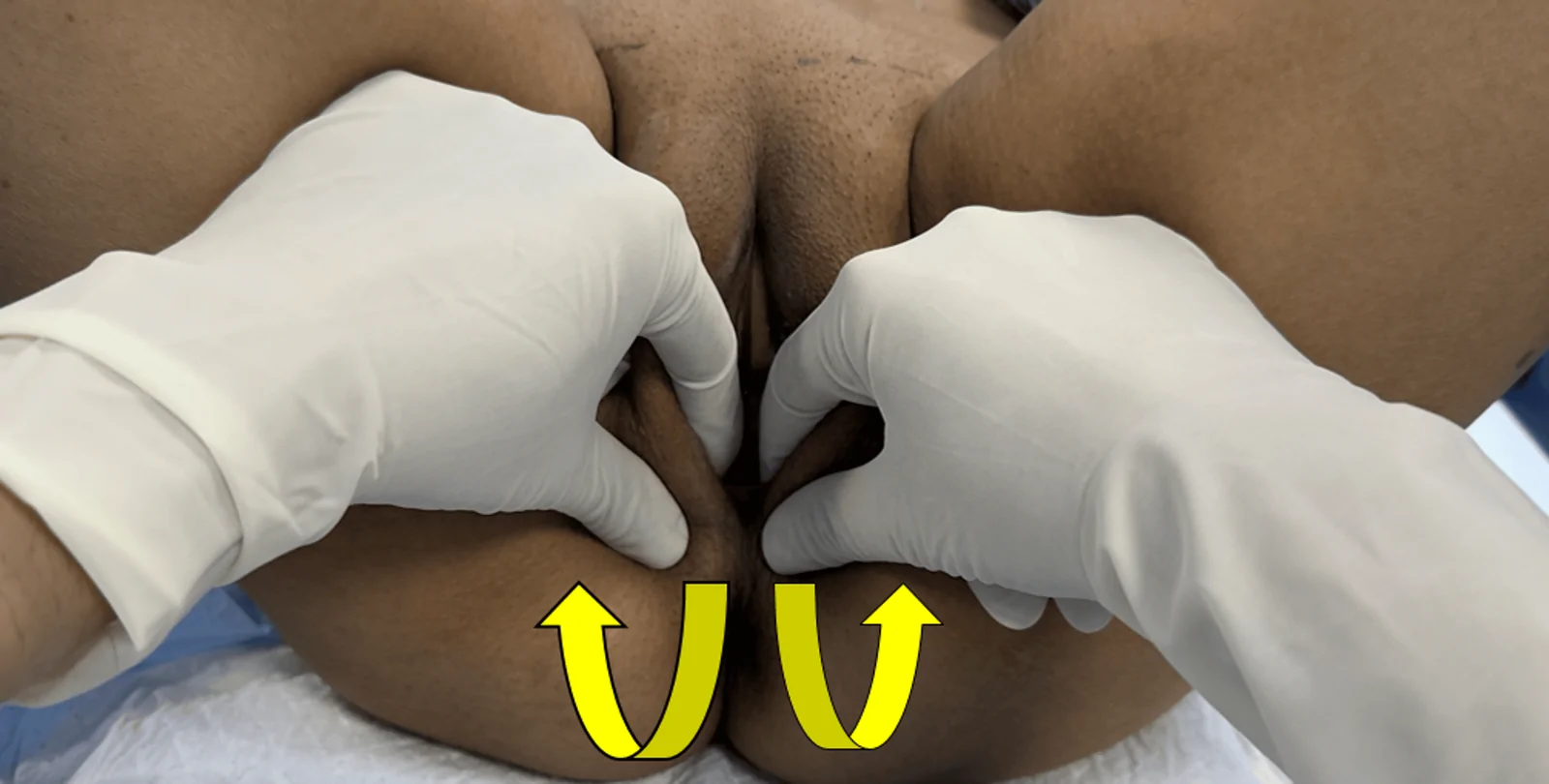
Process of perineal massage. The arrows depict the circular direction of massage done using both fingers.

The process of perineal massage is shown in Figure 2. 

Informed written consent was obtained prior to data collection. Outcomes were assessed by recording the frequency of perineal trauma and the duration of the second stage of labor in both groups. Data were analyzed using descriptive statistics (mean, frequency, percentage, and standard deviation) and inferential statistics, including the t-test for comparing the mean duration of the second stage of labor and the chi-square test for assessing associations with sociodemographic variables, with the level of significance set at p < 0.05.

## Results

The chi-square test was selected because most demographic variables were categorical, and the objective was to determine whether the distribution of these characteristics differed between the experimental and control groups. For variables reported as mean ± SD, the independent-samples t-test was employed to compare group means because the two groups were independent and the variables were measured on a continuous scale. The non-significant p-values indicated that the groups were comparable at baseline, as shown in Table 1. 

**Table 1:** Frequency and percentage distribution of samples on personal and obstetrics variables in experimental and control group. NS: not significant.

Demographic variables	Experimental group (N = 40), f(%)	Control group (N = 40), f(%)	p-value
Age (in years)			
18-22	09(22.5)	13(32.5)	0.792^NS^
23-26	19(47.5)	17(42.5)	
27-30	11(27.5)	09(22.5)	
>30	01(2.5)	01(2.5)	
Mean±SD	24.1±3.14	22.9±2.09	
Education			
No formal education	12(30)	13(32.5)	0.232^NS^
Secondary	13(32.5)	07(17.5)	
Senior secondary	07(17.5)	12(30)	
Graduate	5(12.5)	08(20)	
Post graduate	01(2.5)	-	
Professional	02(05)	-	
Religion			
Hindu	31(77.5)	27(68.7)	0.290^NS^
Muslim	09(22.5)	11(27.5)	
Other	-	02(05)	
BMI (kg/m^2^)			
18-24	20(50)	19(47.5)	0.810^NS^
24-30	20(50)	21(52.5)	
Mean±SD	24±3.5	23.5±3.4	
Type of family			
Nuclear	21(52.5)	20(50)	0.823^NS^
Joint	19(47.5)	20(50)	
Weight of fetus			
1.5-2kg	01(2.5)	-	0.215^NS^
2-2.5kg	15(37.5)	12(30)	
2.5-3kg	19(47.5)	16(40)	
>3kg	05(12.5)	12(30)	
Mean±SD	2.7±33	3.1±37	
Induction of labor			
Spontaneous	09(22.5)	15(37.5)	0.143^NS^
Induced	31(77.5)	25(62.5)	

The chi-square test was used to analyze episiotomy and perineal tears because these variables were categorical (yes/no), and the study aimed to compare the distribution of these outcomes between two independent groups (experimental and control). The test determined whether there was a significant association between group allocation and the occurrence of perineal trauma. Since the data were expressed as frequencies and percentages, the chi-square test was the most appropriate statistical method, as shown in Table 2. A dichotomous categorical variable was compared between two independent groups; therefore, the chi-square test was appropriate. 

**Table 2:** Frequency and percentage distribution of samples on episiotomy and perineal tear among experimental and control group. NS: not significant.

Perineal trauma	Experimental group (N = 40), f(%)	Control group (N = 40), f(%)	Chi-square	p-value
Episiotomy				
No	15(37.5)	09(22.5)	2.143	0.143^NS^
Yes	25(62.5)	31(77.5)		
Perineal tear				
No	36(90)	32(80)	2.635	0.268^NS^
Yes	04(10)	08(15)		

Duration of the second stage of labor was a continuous variable measured in minutes. An independent-samples t-test was used to compare the mean duration between the experimental and control groups. A p-value less than 0.05 was considered statistically significant. The mean duration of the second stage of labor was 33.46 ± 4.35 minutes in the experimental group and 36.72 ± 5.30 minutes in the control group. The difference was not statistically significant (t = -1.32, p = 0.192), indicating that the duration of the second stage of labor was comparable between the two groups. 

**Table 3:** Frequency, percentage and mean duration of second stage of labor among experimental and control group. NS: not significant.

Duration of the second stage of labor in minutes	Experimental group (n = 40)		Control group (n = 40)		t-value	p-value
	f(%)	Mean (SD)	f(%)	Mean (SD)		
<35	24(60)	33.46 (4.35)	18(45)	36.72 (5.30)	-1.32	0.192^NS^
35-45	16(40)		20(50)			
>45	-		02(05)			

## Discussion

The present study was conducted among 80 primigravidae (40 in the experimental group and 40 in the control group) at AIIMS, Jodhpur, to evaluate the effect of perineal priming on perineal trauma and the duration of the second stage of labor. Episiotomy was observed in 62.5% of mothers in the experimental group compared with 77.5% in the control group. Similar findings were reported by Raja et al. [5], who found a lower incidence of episiotomy in the massage group. However, Karaçam et al. [7] and Stamp et al. reported no significant difference between groups. Perineal tears occurred in 10% of mothers in the experimental group and 15% in the control group. Ferreira-Couto et al. [11] reported a higher rate of intact perineum in the intervention group, while Pallavee et al. [5] and Akhlaghi et al. [12] found a higher incidence of tears in the massage group. Karacam et al. [7] found no significant difference in perineal trauma between the two groups. The mean duration of the second stage of labor was lower in the experimental group (33.46 minutes) than in the control group (36.72 minutes). Similar reductions were reported by Pallavee et al. [5], Ferreira-Couto et al. [11], and Akhlaghi et al. [12], whereas Karacam et al. [7] found no difference. Although the reduction in perineal trauma was not statistically significant, the findings suggest a positive trend toward fewer episiotomies, fewer perineal tears, and a shorter second stage of labor among mothers receiving perineal priming. These findings are consistent with previous reviews [10-16]. The lack of statistical significance may be attributed to the small sample size and variations in clinical practices regarding episiotomy use. Perineal priming is a simple, safe, and potentially beneficial intervention.

## Conclusions

In conclusion, the study addressed a clinically relevant issue, as perineal trauma and episiotomy are common concerns among primigravidae and may affect postpartum recovery and quality of life. The intervention, perineal priming and perineal support, is simple, low-cost, and potentially feasible in routine maternity care. It is also regarded as innovative since it included various interventions that had never been combined before. It also creates a sensation of comfort during the labor process and can be performed by women. However, the study also has important methodological limitations. The small sample size may have limited the statistical power to detect meaningful differences. The study was conducted in a single center, which reduces the generalizability of the findings. Moreover, clinical decisions regarding episiotomy may have been influenced by midwives' practices, institutional policies, and individual clinical judgment, which were not fully controlled. The intervention showed a possible positive trend, but stronger evidence from larger randomized controlled trials is needed before routine clinical implementation can be recommended. Thus, the decision could also be based on the midwife's knowledge and the mother's willingness.
